# Rab31 expression levels modulate tumor-relevant characteristics of breast cancer cells

**DOI:** 10.1186/1476-4598-11-62

**Published:** 2012-08-24

**Authors:** Bettina Grismayer, Susanne Sölch, Bastian Seubert, Thomas Kirchner, Sonja Schäfer, Gustavo Baretton, Manfred Schmitt, Thomas Luther, Achim Krüger, Matthias Kotzsch, Viktor Magdolen

**Affiliations:** 1 Clinical Research Unit, Department of Obstetrics and Gynecology, Technical University of Munich, Ismaninger Str. 22, Munich 81675, Germany; 2 Institute of Experimental Oncology and Therapy Research, Technical University of Munich, Ismaninger Str. 22, Munich, 81675, Germany; 3 Medizinisches Labor Ostsachsen, Töpferstr. 17, Bautzen, 02625, Germany; 4 Institute of Pathology, Dresden University of Technology, Fetscherstr. 74, Dresden, 01307, Germany

**Keywords:** rab31, Breast cancer, GTP-binding protein, Intracellular vesicle transport, Proliferation, Adhesion, Invasion, Tumor cell phenotype

## Abstract

**Background:**

Rab proteins constitute a large family of monomeric GTP-binding proteins that regulate intracellular vesicle transport. Several Rab proteins, including rab31, have been shown to affect cancer progression and are related with prognosis in various types of cancer including breast cancer. Recently, the gene encoding rab31 was found to be overexpressed in estrogen receptor-positive breast cancer tissue. In a previous study we found a significant association of high rab31 mRNA expression with poor prognosis in node-negative breast cancer patients. In the present study, we aimed to investigate the impact of rab31 (over)-expression on important aspects of tumor progression *in vitro* and *in vivo*.

**Methods:**

Breast cancer cells displaying low (MDA-MB-231) or no (CAMA-1) endogenous rab31 expression were stably transfected with a rab31 expression plasmid. Batch-transfected cells as well as selected cell clones, expressing different levels of rab31 protein, were analyzed with regard to proliferation, cell adhesion, the invasive capacity of tumor cells, and *in vivo* in a xenograft tumor model. Polyclonal antibodies directed to recombinantly expressed rab31 were generated and protein expression analyzed by immunohistochemistry, Western blot analysis, and a newly developed sensitive ELISA.

**Results:**

Elevated rab31 protein levels were associated with enhanced proliferation of breast cancer cells. Interestingly, weak to moderate overexpression of rab31 in cell lines with no detectable endogenous rab31 expression was already sufficient to elicit distinct effects on cell proliferation. By contrast, increased expression of rab31 in breast cancer cells led to reduced adhesion towards several extracellular matrix proteins and decreased invasive capacity through Matrigel^TM^. Again, the rab31-mediated effects on cell adhesion and invasion were dose-dependent. Finally, in a xenograft mouse model, we observed a significantly impaired metastatic dissemination of rab31 overexpressing MDA-MB-231 breast cancer cells to the lung.

**Conclusions:**

Overexpression of rab31 in breast cancer cells leads to a switch from an invasive to a proliferative phenotype as indicated by an increased cell proliferation, reduced adhesion and invasion *in vitro*, and a reduced capacity to form lung metastases *in vivo*.

## Background

Compartmentalization of eukaryotic cells requires transport of lipids and proteins between distinct membrane-bound organelles. Rab GTPases, which belong to the Ras superfamily of small GTP-binding proteins, are key regulators of membrane trafficking in eukaryotic cells. Up to now, more than 60 different human Rab proteins have been identified [1,2]. The functions of Rab GTPases depend on their ability to alternate between two conformational states, the inactive (GDP-bound) and the active (GTP-bound) state. Furthermore, their function depends on their capacity to reversibly associate with specific membrane compartments [3,4].

A growing number of Rab and Rab-related proteins has been functionally characterized [for a review see 1]. Rab31, also known as rab22B, is a 194 amino acid protein (Mr = 22,000), which shares highest homology with rab22A (71% sequence identity). Rab31 is mainly localized to the trans-Golgi, the trans-Golgi network (TGN) and to endosomes [4,5] and is involved in the vesicle transport from the Golgi apparatus to early and late endosomes, *e.g.* of mannose-phosphate receptors [6]. Furthermore, rab31 was shown to modulate epidermal growth factor receptor (EGFR) internalization in the epidermoid carcinoma cell line A431 [7]. Interaction with GDP/GTP exchange factors (GEFs) such as Gapex-5, EEA-1, and RIN proteins was demonstrated [8,9]. Additionally, direct interaction with the mRNA-binding protein HuR [10] and the phosphatidyl-inositol(PI)-4,5-diphosphate-5-phosphatase ORCL-1 [11] was observed. ORCL-1 plays a key role in the regulation of the levels of PI(4)P and PI(4,5)P_2_, two signaling molecules involved in membrane trafficking and Golgi/TGN organization [11].

Dysfunctions in Rab pathways can lead to immunodeficiencies and neurological disorders. Also, dysregulation of Rab expression was shown to affect cancer progression [12,13]. Rab31 was identified as one out of 11 genes that are overexpressed in estrogen receptor (ER)-positive breast cancer patients [14]. Recently, we could show, that elevated rab31 mRNA levels are significantly associated with shorter distant metastasis-free and overall survival of untreated, lymph node-negative breast cancer patients [15]. More extensively characterized Rab proteins such as rab25 and rab21 were shown to be associated with increase in tumor cell proliferation and are required to promote cancer cell invasion [13,16]. High expression levels of rab25 are present in breast and ovarian cancer tissues and are associated with poor outcome of the patients [17]. Conversely, rab25 overexpression in cancer cell lines substantially suppressed cell invasion *in vitro* and tumor formation *in vivo*[18]. This indicates that dysregulation of Rab expression may exert either tumorigenic or tumor suppressive effects [12].

However, little is known about the tumor biological effects of rab31 expression in breast cancer. Therefore, in the present study we aimed at analyzing the impact of differential rab31 expression in breast cancer cells on important aspects of tumor progression *in vitro* and *in vivo*. Using proliferation, adhesion, and invasion assays, we first characterized the phenotype of the cell transfectants with different rab31 expression levels *in vitro*, and then monitored the impact of rab31 expression on experimental metastasis in a xenograft tumor model in mice. Increased rab31 protein levels were associated with enhanced proliferation of breast cancer cells, led to a reduced adhesion of cells towards extracellular matrix proteins and decreased invasive capacity through Matrigel^TM^. In addition, we observed a significantly impaired metastatic dissemination of rab31 overexpressing MDA-MB-231 cells to the lung using a xenograft mouse model. Taken together, our results demonstrate that rab31 overexpression leads to a switch from an invasive to a proliferative phenotype as indicated by an increased cell proliferation, reduced cell adhesion, and decreased cellular invasion *in vitro* and *in vivo*.

## Results

### Generation and characterization of polyclonal antibodies directed to rab31

**Figure 1:**
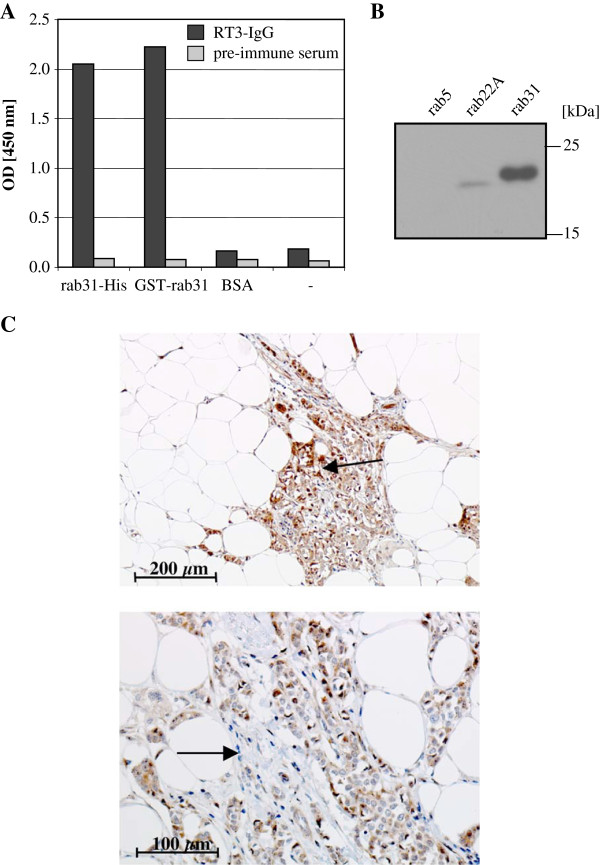
**Characterization of polyclonal antibodies directed to human rab31. (A)** The reaction pattern of polyclonal antibody (pAb) RT3-IgG (black) and of the pre-immune rabbit serum (gray) was analyzed by a ‘one-sided ELISA’ assay [19]. rab31-His, purified recombinant, histidine-tagged rab31, used for immunization; GST-rab31, purified recombinant GST-rab31 fusion protein; BSA, bovine serum albumin. **(B)** In Western blot analyses, pAb RT3-IgG strongly reacts with recombinant rab31 and not at all or only weakly with the highly homologous Rab proteins rab5 and rab22A, respectively. **(C)** Immunohistochemical staining of paraffin-embedded, formalin-fixed breast cancer specimens with pAb RT4-IgG. Specific immunostaining is observed in the cytoplasm as well as in the nucleus of cancer cells (upper panel, see arrow), stromal cells are stained less frequently (lower panel, see arrow).

For immunization of rabbits and chickens, purified human rab31 carrying an N-terminal histidine (His)_6_-tag was used and the generated polyclonal antibodies directed to rab31 (two derived from chicken, and four from rabbits) subsequently tested for specificity. The reaction pattern of the IgG-fraction of the most suitable antibody from rabbit #3, pAb RT3-IgG, by ‘one-sided ELISA’ [19] and Western blot is depicted in Figure 1A and B. Using the ‘one-sided ELISA’ assay, in which microtiter plates were coated with rab31 antigen or unrelated control protein, we found a strong reaction of pAb RT3-IgG with its immunogen rab31-His as well as with recombinant GST-rab31 (which consists of human rab31 fused C-terminally to the bacterial glutathione *S*-transferase). No reaction was observed when wells were coated with the control protein BSA instead of rab31 (Figure 1A). Next, we analyzed whether pAb RT3-IgG may cross-react with other members of the Rab protein family that are closely related to rab31 such as rab5 and rab22A. With exception of the C-terminal part (≈ 30 amino acids), which harbors important residues for the interaction with Rab-binding proteins involved in C-terminal prenylation [20], rab5 and rab22A are highly homologous to rab31: within the N-terminal 167 residues, rab31 shares 85 identical plus 60 homologous amino acids with rab5 (= 86.8% homology), and even 133 identical plus 20 homologous amino acids with rab22A (= 91.6% homology). To test for cross-reactions, equal amounts of recombinant human rab5, rab22A, and rab31 protein were applied in Western blot analysis. Here, for rab5 no cross-reaction was observed; for rab22A, a slight reaction was observed only after extended exposure of the blot (Figure 1B). Furthermore, by immunohistochemical analyses of breast cancer tissue sections, both pAb RT3-IgG and pAb RT4-IgG (from animal #4) were qualified for detection of rab31. As depicted in Figure 1C, rab31 immunostaining was mainly observed in the cytoplasm of tumor cells with occasional perinuclear/nuclear staining. Less frequently, elongated fibroblast-like cells within the stroma were stained. 

### Generation and characterization of breast cancer cell lines overexpressing rab31

First, various breast cancer cell lines were analyzed by quantitative PCR for endogenous rab31 mRNA expression levels [15,21]. Highest rab31 mRNA expression was found in ZR75 cells (ratio 33.4 zmol rab31 mRNA per zmol TATA box-binding protein [TBP] mRNA, which was used as a housekeeping gene for normalization). MDA-MB-231 and adriamycin-resistant MCF-7 cells expressed moderate rab31 mRNA levels (ratio 20.2 and 24.0 rab31/TBP, respectively), whereas MDA-MB-435 and CAMA-1 cells did not express any detectable rab31 mRNA. Therefore, for further experiments, we selected MDA-MB-231 cells displaying a basal endogenous rab31 expression and the two rab31-negative cell lines.

**Figure 2:**
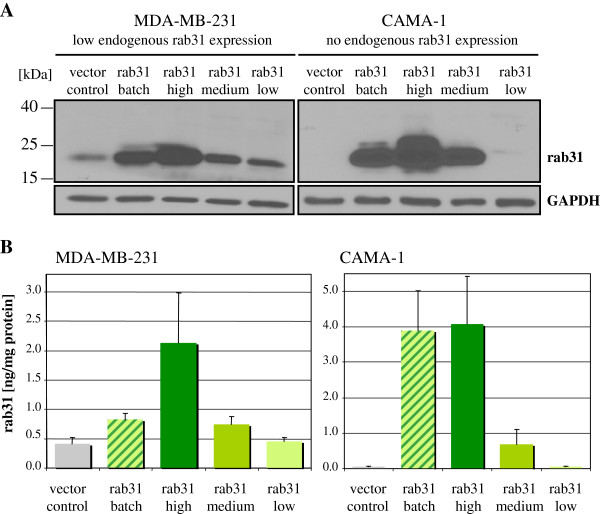
**Rab31 expression levels in stably transfected breast cancer cells. (A)** pAb RT3-IgG specifically detects rab31 in cell lysates from stably transfected MDA-MB-231 and CAMA-1 breast cancer cells in Western blot analyses. Vector control, MDA-MB-231, or CAMA-1 cells were stably transfected with the empty vector pRcRSV; batch, parental MDA-MB-231 (low endogenous rab31 expression) or CAMA-1 (no endogenous rab31 expression) cells were stably transfected with the pRcRSV-based rab31 expression vector; high, medium or low, selected cell clones of batch-transfected MDA-MB-231 or CAMA-1 cells with differing high, medium or low protein levels of rab31. **(B)** Rab31 antigen values (ng/mg of total protein) in rab31-transfected MDA-MB-231 and CAMA-1 cells, determined by rab31-ELISA. Mean values (± SEM) of at least three independent experiments are depicted.

The expression pattern of rab31 in breast cancer cell lines MDA-MB-231, CAMA-1, and MDA-MB-435 upon stable transfection with the eukaryotic expression plasmid pRcRSV harboring the rab31 cDNA sequence was initially analyzed by Western blot analysis. In all cell lines tested, an increased rab31 protein expression was monitored (apparent molecular weight of about 22 kDa; Figure 2A). MDA-MB-231 cells transfected with the empty expression vector pRcRSV (vector control) - in line with the PCR results - displayed basal endogenous rab31 protein expression whereas CAMA-1 (and MDA-MB-435; data not shown) vector control cells did not display any reactive protein (Figure 2A). In addition, in the case of MDA-MB-231 and CAMA-1 cells, cell clones were isolated from the batch transfectants by two rounds of subcloning in order to get individual cell lines expressing homogenous high, medium or low levels of rab31 protein (Figure 2A).

In addition to Western blot analysis, rab31 protein levels in cell lysates were quantified by our own rab31-specific ELISA, employing the commercial mAb M01 (Novus Biologicals) as catcher antibody and pAb RT3-IgG for detection. Rab31 protein values determined in cell lysates of MDA-MB-231 and CAMA-1 vector control and batch cells, and in isolated cell clones were very similar to the expression levels monitored by Western blot analysis: highest rab31 antigen values were observed in the rab31 high expressing MDA-MB-231 and CAMA-1 cell clones (2.13 ng/mg, and 4.08 ng/mg protein, respectively) whereas low/no rab31 antigen was found in low expressing clones (0.46 ng/mg, and <0.10 ng/mg protein, respectively; Figure 2B).

Transfected cells were also characterized by immunocytofluorescence [22]. The intensity of rab31 immunocytostaining of transfected cells was in parallel with the results obtained by Western blot analysis (data not shown).

### Proliferative characteristics of breast cancer cells overexpressing rab31

**Figure 3:**
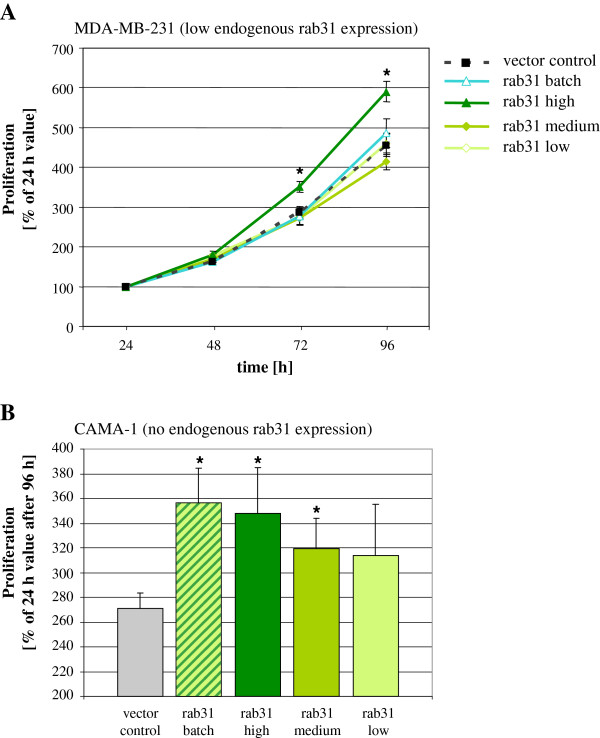
**Rab31 overexpression enhances proliferation of breast cancer cells *in vitro. *** Cells were seeded in triplicate onto 24-well plates, detached with 0.05% EDTA-solution after 24, 48, 72 and 96 h of cultivation and counted with a Neubauer-chamber under trypan blue exclusion. Cell number at 24 h was set to 100%; increase in cell number was expressed relative to the 24 h value in %. Mean values (± SEM) of at least three independent experiments are depicted. Statistically significant differences (p < 0.05) to the vector control group are indicated by an asterisk. **(A)** Only MDA-MB-231 cell clones with a homogenous high rab31 expression show a significant increase in cell proliferation compared to vector control cells. **(B)** CAMA-1 batch-transfected cells, as well as CAMA-1 cell clones with homogenous medium and high expression of rab31, respectively, display a significant increase in cell proliferation compared to vector control cells after 96 h of cultivation.

The impact of rab31 overexpression on tumor cell proliferation was analyzed by manual cell enumeration. MDA-MB-231 batch-transfected cells and single MDA-MB-231 cell clones with low or medium rab31 expression showed no significant differences in cell growth rate. Only cells highly overexpressing rab31 (approximately 5-fold higher than basal endogenous rab31 levels in the vector control cell line; see Figure 2B) showed significantly enhanced cell proliferation after 72 and 96 h of culturing (p < 0.05; Figure 3A). To exclude clonal effects, we analyzed various cell clones with either high, medium or low rab31 expression levels to confirm that cell clones with similar protein levels of rab31 behave similarly with respect to cell growth (data not shown).

A comparable effect of rab31 overexpression on cell growth was monitored in CAMA-1 cells. In contrast to MDA-MB-231 cells, in transfected CAMA-1 cells (parental CAMA-1 cells do not endogenously express rab31) already low protein levels of rab31 seem to enhance cell growth compared to vector control cells (Figure 3B). Batch-transfected cells, which show an overall moderate overexpression of rab31, as well as medium and high expressing CAMA-1 cell clones were characterized by significantly enhanced proliferation 96 h after cell plating (Figure 3B). Analysis of additional, individual cell clones expressing high, medium, or low rab31 protein levels again confirmed similar growth rates of cell clones with similar levels of rab31 (data not shown). In FACS analyses by employing annexin V staining, we found no indication for any differences in apoptosis between vector control and rab31 high overexpressing cells, neither in MDA-MB-231 nor in CAMA-1 cells (data not shown).

Additionally, the human MDA-MB-435 cell line was stably transfected with the rab31 expression plasmid. Whether this cell line is derived from breast cancer or from melanoma has been discussed controversial. Recent results, however, indicate that this cell line should be considered as a poorly differentiated, aggressive breast tumor cell line, with expression of both epithelial and melanocytic markers [23]. Alike CAMA-1 cells, parental MDA-MB-435 cells do not endogenously express rab31. Similarly, we observed that proliferation of MDA-MB-435 cells was significantly enhanced in rab31 batch-transfected cells compared to vector control cells (data not shown).

### Adhesion of breast cancer cells to extracellular matrix proteins

**Figure 4:**
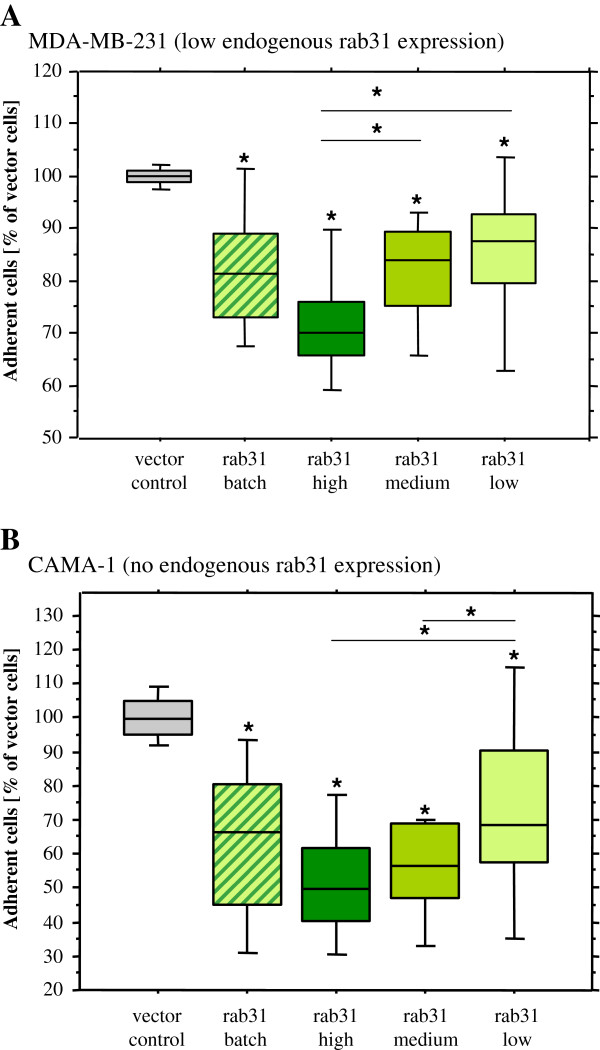
**Rab31 overexpression reduces the adhesive capacity of human breast cancer cells *in vitro. *** Stably transfected MDA-MB-231 cells **(A)** and CAMA-1 cells **(B)** were seeded on collagen type IV-coated microtiter plates. After 2 h of cell cultivation, the number of adherent cells was monitored by the hexosaminidase activity assay. At least 5 independent experiments were performed in triplicates each. The results are given in % relative to the cell number of adherent vector-transfected control cells. Whisker box plots indicate the 25th and 75th percentile, the vertical bars indicate the 10th and 90th percentile. The median value of at least 5 experiments is indicated by a bar within the box. Statistically significant differences (p < 0.05) to the vector control group are indicated by an asterisk, differences between other groups are indicated by a line with an asterisk.

In order to analyze the effect of rab31 overexpression on tumor cell adhesion, rab31-transfected breast cancer cells were incubated on polystyrene plates coated with different extracellular matrix (ECM) proteins. Towards collagen type IV (Figure 4A), batch-transfected MDA-MB-231 cells overexpressing rab31 showed moderate but significantly reduced adhesion (reduction of 17.7%, p < 0.05). When analyzing individual MDA-MB-231 cell clones, we observed the strongest decrease of cellular adhesion in rab31 high expressing cells (reduction of 28.0%, p < 0.05). For cells with medium rab31 expression, a moderate reduction (17.6%, p < 0.05) in adhesive capacity was observed. Low rab31 expressing cells displayed less pronounced but still significantly reduced adhesion (14.4%; p < 0.05) towards collagen type IV. Thus, decrease in cellular adhesion of the transfected MDA-MB-231 cells to collagen type IV depends on the extent of the rab31 protein level (Figure 4A). Likewise, similar results were obtained for the ECM proteins fibronectin, laminin and collagen type I: overexpression of rab31 led to significantly reduced adhesive capacities of the transfected MDA-MB-231 cells to each of the ECM proteins being most pronounced in the rab31 high expressing cell clones (Table 1). Analysis of additional individual cell clones with similar (high, medium or low) rab31 expression levels showed a comparable adhesive capacity towards ECM proteins investigated (data not shown).

**Table 1:** **Rab31 overexpression reduces the adhesive capacity of human breast cancer cells *in vitro ***

	**rab31 batch**	**rab31 high**	**rab31 medium**	**rab31 low**
Col IV	82.3+/-4.0*	72.0+/-3.1*	82.4+/-4.6*	85.6+/-5.0*
Col I	84.8+/-3.0*	70.5+/-2.7*	87.4+/-4.2*	81.6+/-5.8*
Fn	84.7+/-3.2*	71.6+/-3.0*	93.4+/-7.1*	95.8+/-4.7*
Vn	82.3+/-3.3*	63.1+/-3.3*	83.8+/-4.3*	90.2+/-4.9*
Ln	81.4+/-5.4*	67.7+/-4.4*	91.1+/-8.6*	88.2+/-11.1*

MDA-MB-231 cells were seeded on 96-well plates coated with the extracellular matrix proteins collagen type I (Col I), collagen type IV (Col IV), fibronectin (Fn), vitronectin (Vn) or laminin (Ln). After 2 h of cell cultivation, the number of adherent cells was monitored by the hexosaminidase activity assay. At least 5 independent experiments were performed in triplicates each. The results are given in % relative to the cell number of adherent vector–transfected control cells. The mean values +/- SEM are depicted. Statistically significant differences (p < 0.05) to the vector control group are indicated by an asterisk.

The observed reduced adhesive capacity was not restricted to rab31 overexpressing MDA-MB-231 cells since expression of rab31 in CAMA-1 cells reduced cell adhesion to various ECM components in a similar manner. Batch-transfected rab31 expressing CAMA-1 cells showed a moderate but significant reduction of adhesion (36.4%; p < 0.05) towards collagen type IV, compared to vector control cells. CAMA-1 cells with high expression of rab31 showed the strongest reduction in cell adhesion (48.5%; p < 0.05). Yet, cells with moderate rab31 overexpression also displayed significantly reduced adhesion (45.1%; p < 0.05). Even low rab31 expressing cell clones were characterized by a still significant reduction in adhesion to collagen type IV (27.3%; p < 0.05), compared to vector control cells (Figure 4B). Cell adhesion assays performed with the various stably transfected CAMA-1 cell lines on plates coated with collagen type I and vitronectin revealed similar results (data not shown).

### Characterization of the invasive potential of breast cancer cells overexpressing rab31

**Figure 5:**
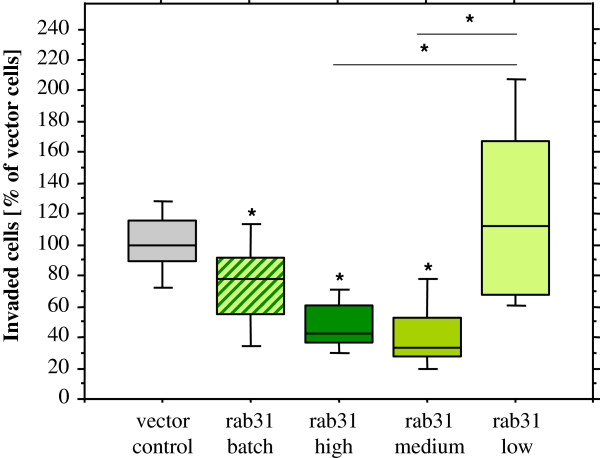
**Rab31 overexpression reduces the invasive capacity of cells *in vitro. *** Stably transfected MDA-MB-231 cells were seeded into the upper compartments of Matrigel™-coated invasion chambers. After 24 h of incubation, invaded cells were fixed, stained, and counted. At least three independent experiments were performed in triplicates each. Results are given in %, normalized to the number of invaded vector-transfected cells. Whisker box plots indicate the 25th and 75th percentile, the vertical bars indicate the 10th and 90th percentile. The median value of at least three independent experiments is indicated by a bar within the box. Statistically significant differences (p < 0.05) to the vector control group are indicated by an asterisk, differences between other groups are indicated by a line with an asterisk.

Next, the invasive properties of rab31 overexpressing MDA-MB-231 cells were investigated in a Matrigel^TM^ invasion assay and compared to those of vector control cells. Batch-transfected MDA-MB-231 cells showed a moderate but significantly reduced invasive capacity through the extracellular matrix (25.3%; p < 0.05). Strikingly, MDA-MB-231 cell clones overexpressing high or medium rab31 protein levels displayed strongly impaired invasion (53.1% and 58.5% reduction, respectively; p < 0.05). On the contrary, the invasive capacity of the rab31 low expression cell clone was comparable to that of the vector control cells (Figure 5). Parental CAMA-1 cells do not display any invasion through Matrigel^TM^, thus, we did not analyze this cell line for effects of rab31 overexpression in this cell biological assay.

### Effects of rab31 overexpressing breast cancer cells on lung metastasis in a xenograft mouse-model

**Figure 6:**
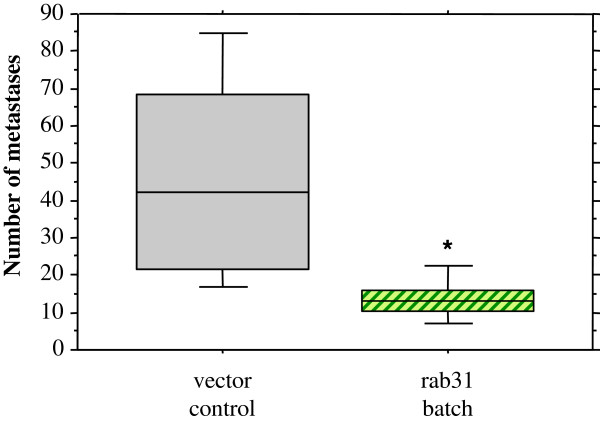
**Overexpression of rab31 affects lung colonization and metastatic growth of human breast cancer cells.** Stably transfected, *lacZ*-tagged, MDA-MB-231 cells were inoculated into nude mice via tail vein injection. Mice received either vector control cells (grey column, n = 7) or batch-transfected rab31 cells (green column, n = 7). Animals were sacrificed at day 35 after injection, lungs were collected and stained with X-Gal. Metastases were counted in lungs of the mice. Whisker box plots indicate the 25th and 75th percentile, the vertical bars indicate the 10th and 90th percentile. Results are expressed as the median number of metastases. The median value is indicated by a bar within the box. Statistically significant differences (p < 0.05) to the vector control group are indicated by an asterisk.

In order to explore the impact of rab31 overexpression in breast cancer cells on the capacity of these cells to form lung metastases *in vivo*, batch-transfected MDA-MB-231 cells tagged with the *lacZ*-gene were injected into the tail veins of immune-compromised female nude CD1 *nu/nu* mice. Mice were sacrificed 35 days after tumor inoculation and lungs removed for X-Gal staining. Blue colonies (indicating metastases) were identified on the surface of the lungs and counted. The number of lung metastases was dramatically and significantly reduced in mice which were inoculated with cells overexpressing rab31 (n = 7; median: 13 colonies) compared to mice injected with vector control cells (n = 7; median: 42 colonies) (Figure 6).

## Discussion

Rab proteins constitute the largest family of monomeric small GTPases. Numerous studies have established that Rab proteins are distributed to distinct intracellular compartments and regulate transport between organelles. Mutations in Rab proteins as well as altered GTPase expression or activity were shown to be involved in neurologic and neurodegenerative diseases, lipid storage disorders and cancer [24]. A growing number of Rab proteins such as rab5, rab11, rab21, rab25, and rab27B have been shown to be associated with tumor growth/behavior and prognosis of breast cancer patients [16,25,26]. However, still few data are available on rab31 expression in cancer in general or its impact on disease progression in particular [10,27]‒ [30]. Abba et al. [14] described that rab31 mRNA is overexpressed in ERα-positive breast cancer tissue. We recently reported that elevated rab31 mRNA expression in primary tumor tissue is associated with poor prognosis of lymph node-negative breast cancer patients [15].

In the present study, antibodies were raised against purified recombinant human rab31 and tested for cross reactivity with other, closely related Rab proteins, rab5 and rab22A. Because the generated polyclonal antibodies turned out to be highly specific for rab31, rab31 protein expression was verified in breast cancer tissue by immunohistochemistry. We detected a weak to moderate cytoplasmic staining for rab31 and, occasionally, strong perinuclear and/or nuclear staining of cancer cells, whereas stromal cells were less frequently stained. This cellular staining pattern is in accordance with data published in the human protein atlas (see http://www.proteinatlas.org) in normal as well as tumor tissue. Moreover, in immunocytochemistry, we occasionally observed a pronounced perinuclear staining in MDA-MB-231 cells overexpressing rab31 which is very similar to the results of published immunocytochemical studies showing in different cell types a perinuclear patch of rab22B/rab31 in the trans-Golgi region [5,7,8; Additional file 1: Figure S1].

To analyze possible cellular effects of variable rab31 protein expression, we stably transfected breast cancer cell lines with the eukaryotic expression plasmid pRcRSV harboring the rab31 cDNA sequence. In general, data based on unphysiologically high protein overexpression should be interpreted with caution. However, applying Western blot analysis and, in particular, a newly developed sensitive ELISA for quantitation of rab31, we found that for instance in MDA–MB-231 cells rab31 protein was increased up to about 5-fold only in the high expressing clones (≈ 2 ng/mg total protein) as compared to vector control cells (≈ 0.4 ng/mg total protein). In a study by Bao et al. [31], the physiological rab31 content in platelets was estimated to be approximately 5 ng/mg total protein. Thus, it can be concluded that in the present study modulation of rab31 levels was within the physiologic range.

On one hand, overexpression of rab31 caused enhanced cell growth of breast cancer cells. These effects are not related to an increase in apoptosis in vector control cells if compared to rab31 overexpressing cells. Interestingly, using cell lines with a basic level of endogenous rab31 expression (MDA-MB-231), high levels of rab31 were necessary to induce a significant increase in cell proliferation. In opposite, already moderate levels of rab31 significantly elevated proliferation in cell lines not displaying any detectable endogenous rab31 expression (CAMA-1 and MDA-MB-435). On the other hand, high levels of rab31 in breast cancer cells resulted in significantly reduced adhesion towards ECM proteins as well as decreased invasive capacity. Likewise, the effects on cell adhesion and invasion were dose-dependent since effects monitored in low rab31 expressing cell clones differed significantly from those observed in rab31 high expressing cell clones. Comparing cell lines with differential endogenous rab31 levels, again the strongest effects were detected in breast cancer cell lines not showing any detectable endogenous rab31 expression (CAMA-1 and MDA-MB-435). In line with its reduced invasive capacity in Matrigel^TM^ invasion assays, we observed a significantly reduced number of lung metastases in immunologically compromised mice injected with rab31 overexpressing MDA-MB-231 cells compared to mice injected with vector control cells. Since development and growth of tumor metastases require that neoplastic cells must either have the potential to shift between proliferative and invasive phenotypes or simply express both simultaneously [32], our results suggest that overexpression of rab31 may lead to a proliferative rather than an invasive phenotype in breast cancer cells (Table 2). 

**Table 2:** Characteristics of MDA-MB-231 cells expressing different levels of rab31

	**rab31 Expression**	
	**Low**	**High**
Proliferation	+	++
Adhesion	++	+
Invasion	++	+
Lung colonization	++	-

Rab31, depending on its expression level, modulates the switch between a more proliferative *versus* invasive phenotype in MDA-MB-231 breast cancer cells.

To verify that the observed cellular effects are not artefacts due to overexpression of a protein *per se*, we also analyzed overexpression of two rab31 mutants in MDA–MB-231 cells: rab31-Q64L is equivalent to constitutively activated GTPase-deficient mutants in other Rab proteins [33], whereas rab31-ΔCC lacks the two C-terminal cysteine residues obligatory for prenylation (responsible for membrane association) and, in consequence, is inactive. In fact, overexpression of rab31-Q64L in MDA-MB-231 cells, similar to wild-type rab31 overexpression, led to significantly increased cell proliferation and decreased adhesion to ECM proteins, whereas cells overexpressing the inactive rab31-ΔCC behaved like the vector control cells (Additional file 2: Figure S2).

Recently, it was shown that rab31 levels are regulated by the mRNA binding protein HuR [10]. HuR is aberrantly expressed in early stages of breast carcinogenesis and high cytoplasmic expression is associated with reduced patients' survival in breast cancer [34,35]. Through its post-transcriptional influence on specific target mRNAs, HuR modulates cellular response to proliferative, apoptotic, differentiation and other stimuli [reviewed in [36]. By enhancing the stability of rab31 transcripts and regulation of their translation, HuR may cause higher rab31 levels in tumor cells. In fact, when HuR expression was silenced in epithelial 184B5Me breast cancer cells, a significant reduction of rab31 mRNA expression was observed [10]. Consistent with published results for other cancer types, HuR overexpression in MDA-MB-231 breast cancer cells (also used in the present study) resulted in increasing cellular growth rates and alterations in cell cycle kinetics *in vitro*[37]. Surprisingly, however, HuR overexpression significantly supressed tumor growth *in vivo* whereas vector control and wild-type MDA-MB-231 cells grew similarly and resulted in much larger tumors than those formed by HuR overexpressing cells. This is very similar to our observation, indicating a strongly reduced metastatic capacity of rab31 overexpressing cancer cells in a xenograft mouse model despite the fact that overexpression of rab31 leads to increased proliferation of MDA-MB-231 cells *in vitro* and high rab31 mRNA levels in (highly proliferating) primary tumor tissue are associated with a poor prognosis in breast cancer patients [15].

In contrast to other Rab proteins [38], the molecular mechanism of rab31 function is still unclear, in spite of identification of cytoskeletal participation and several interacting proteins. Immunocytochemistry analyses indicate that a large fraction of rab31 protein is localized to the perinuclear region, in the TGN and in endosomes [4,7,8]. Recently, it has been demonstrated that rab31 is involved in the transport of mannose 6-phosphate (Man-6-P) receptors (MPRs), especially of the cation-dependent (CD-)MPR, from the TGN to endosomes [6,9].

In eukaryotic cells, MPRs are key components of the lysosomal enzyme targeting system that bind newly synthesized Man-6-P-containing acid hydrolases and function as efficient cargo transporters from the TGN to endosomal/lysosomal compartments [39,40]. The two distinct MPRs, the 46 kDa CD-MPR and the 300 kDa cation-independent (CI-)MPR are the sole members of the P-type lectin family [reviewed in [41]. Although CD-MPR and CI-MPR bind the same array of proteins, the respective affinity of each MPR for different phosphorylated glycoproteins varies. This provides a biochemical mechanism, which, in part, may explain the interaction of the two MPRs with overlapping yet distinct subsets of ligands *in vivo*[42]. The CI-MPR has been described to bind proteins bearing the Man-6-P recognition marker as well as the peptide hormone IGF-II and to be implicated in numerous cellular processes, including cell growth, apoptosis, and cell migration [43,44]. In addition, CI-MPR has been shown to interact also with a number of proteins that do not contain Man-6-P such as urokinase-type plasminogen activator receptor (uPAR) [45]‐ [47]. The uPAR binding epitope on CI-MPR is different from those binding Man-6-P and IGF-II. Binding of uPAR to MPRs seems to be specific for CI-MPR and independent of its ligand, the serine protease uPA. CI-MPR was proposed to be involved in the turnover of uPAR and to regulate the cell surface concentration of uPAR by directing uPAR to lysosomes as well as internalizing uPAR when it interacts with uPA [45]. The detection of uPAR in endosomes agrees well with the primary function of MPRs, which is the transport of ligands from the TGN or cell surface to the endosome. Interestingly, both uPA and uPAR were upregulated in CI-MPR knockdown cells [48].

Several splice variants of human uPAR have been described and their expression was analyzed in breast cancer cells and tissues. One of these splice variants, uPAR-del4/5, encodes a molecular form of uPAR which lacks domain DII of uPAR and does not interact with its ligand uPA [21,22]. Quantification of mRNA levels of uPAR-del4/5 in tumor tissues revealed that higher uPAR-del4/5 expression - similar to rab31 - is associated with shorter disease-free survival of breast cancer patients [15,21,49]. Since uPAR as well as rab31 mRNA are known to be regulated by the RNA binding protein HuR both *in vitro* and *in vivo*[10,50], the observed elevated uPAR-del4/5 as well as rab31 expression in metastasizing breast cancer may be due to increased HuR expression in these tumors and points to a possible link between rab31 and the uPA/uPAR system in cancer cells. All three factors, HuR, uPAR-del4/5, and rab31, have been demonstrated to modulate cellular processes such as proliferation, adhesion and/or invasion. Strikingly, although high expression levels of these three factors (measured in highly proliferating primary tumor tissue) are clearly associated with poor prognosis of breast cancer patients, in experimental animal xenograft tumor models - using the same breast cancer cell line MDA-MB-231 - overexpression in each case unexpectedly leads to a seemingly reduced tumorigenicity. Tumor progression is known to be a multi-step process, including transitions of the malignant phenotype of tumor cells from a predominantly proliferative to a mainly invasive phenotype as well as interactions between tumor cell and tumor-associated stromal cells. The results obtained with the xenograft animal models, therefore, may not directly mirror the malignant features of rab31-, uPAR-del4/5- or HuR overexpressing tumors in human breast cancer. With regard to the plasticity of tumor cells in reliance of rab31 expression, our results, however, demonstrate that rab31 expression is implicated in modulation of tumor-relevant biological processes.

## Conclusions

During tumor progression, cancer cells sequentially acquire different malignant phenotypes: in primary tumors, cell proliferation and angiogenesis are induced; subsequently, the invasive capacity of the tumor cells as well as their motility is stimulated to generate micrometastases; finally, the proliferation phenotype has to be recovered to trigger growth of the metastatic foci. In breast cancer cells, overexpression of the GTP-binding protein rab31 (which is involved in intracellular trafficking) leads to a switch from an invasive to a proliferative phenotype as indicated by increased cell proliferation, reduced adhesion and invasion, and a reduced capacity to form lung metastases.

## Methods

### Cell culture and cell transfection

The human breast adenocarcinoma cell lines MDA-MB-231 and CAMA-1 (American Type Culture Collection [ATCC], Manassas, VA) were cultured in Dulbecco’s modified Eagle medium (DMEM) (Gibco BRL, Eggstein, Germany) supplemented with 10% (v/v) fetal calf serum (FCS) (Gibco BRL), 10 mM 4-(2-hydroxyethyl)-1-piperazineethanesulfonic acid (HEPES) (Gibco BRL), 0.55 mM L-arginine, and 0.272 mM L-asparagine (Sigma-Aldrich, Saint-Louis, MO). In some experiments, the human MDA-MB-435 cell line (ATCC) was used as well. Cells were routinely checked by polymerase chain reaction (PCR) to be free of mycoplasma infection.

Human full-length rab31 cDNA was obtained by real-time PCR (RT-PCR) using mRNA from human breast cancer tissue as a template. The cDNA was directionally subcloned into the pRcRSV plasmid using 5´-HindIII and 3´-XbaI restriction sites, which were introduced into the cDNA by PCR. The sequence was verified by sequencing and was found to be identical to that of the Genbank entry NM_006868.3.

Cells were transfected using Lipofectin® (Invitrogen, Karlsruhe, Germany). Cell transfectants were selected by addition of 1 mg/ml G418 (Gibco BRL) to the cell culture medium (Gibco BRL). In an independent transfection experiment, cell clones with a homogenous high, medium, or low expression of rab31 were isolated by limited dilution.

### Generation of polyclonal antibodies directed against rab31

Recombinant rab31, harboring an N-terminal histidine (His)_6_-tag, was expressed in *Escherichia coli* and purified by nickel-nitrilotriacetic acid agarose affinity chromatography (Qiagen, Hilden, Germany) under denaturing/slightly reducing conditions as described previously [51]. The purified recombinant rab31 protein, dialyzed against phosphate-buffered saline (PBS), pH 7.4, containing 1 mM dithiothreitol (DTT), was used as antigen for immunization of chickens and rabbits (Pineda Antibody Service, Berlin, Germany). Chicken IgY antibodies were purified from egg yolk as previously described [52]. The IgG fraction of polyclonal rabbit antibodies was isolated from hyperimmune serum of rabbits by protein A affinity chromatography (Pineda Antibody Service).

### One-sided ELISA for testing the reactivity of rab31-directed polyclonal antibodies

Purified antibodies were characterized using a ‘one-sided ELISA’ assay in which the specific antigen or an irrelevant protein were coated onto the wells of polystyrene microtiter plates as described [19]. Briefly, test antigens diluted to 1 μg/ml in coating buffer (67 mM Na_2_CO_3_, 100 mM NaHCO_3_, pH 9.6) were adsorbed to the wells of 96-well microtiter plates (Thermo Scientific, Nunc, Rochester, NY) and incubated overnight at 4°C. Following three washing steps with Tris-buffered saline (TBS), pH 7.4, containing 0.05% (v/v) Tween-20 (TBS-T), plates were blocked using blocking buffer (TBS-T containing 2% [w/v] bovine serum albumin, albumin [BSA], Sigma-Aldrich) for 1 h at room temperature (RT). After washing, polyclonal rabbit or chicken antibodies to rab31 (diluted 1:5,000 in TBS-T containing 0.5% [w/v] BSA) were applied to the wells for 1 h at RT. Subsequently, incubation with a secondary horseradish peroxidase (HRP)-conjugated goat anti-rabbit IgG (Jackson ImmunoResearch Lab, West Grove, PA) or an HRP-labeled rabbit anti-chicken IgY (Sigma-Aldrich) as detection antibodies was carried out for 2 h at RT. Antigen binding was visualized using 3,3’5,5’-tetramethylbenzidine (TMB) / H_2_O_2_ as substrate (Thermo Scientific, Pierce, Stonehouse, UK) for 20 min at RT. After color development, the reaction was stopped by addition of 0.5 M H_2_SO_4_. The optical density of the resulting color was determined at 450 nm using an automated ELISA plate reader.

### Extraction of proteins from breast cancer cells for Western blotting and ELISA

Cultured MDA-MB-231 and CAMA-1 cells were washed with PBS, pH 7.4 (Invitrogen), and cell pellets disrupted by two freezing and thawing cycles, followed by solubilization of rab31 antigen in extraction buffer (20 m M Tris-HCl, 125 mM NaCl, pH 7.6, containing 1% [v/v] Triton X-100, and the “Complete” protease inhibitor cocktail, Sigma-Aldrich). Cell lysates were centrifuged at 13,000 x *g* for 10 min at 4°C and the supernatant collected. The protein content was quantified using the Pierce Micro BCA^TM^ protein assay reagent kit (Thermo Scientific, Pierce).

### Western blot

Proteins were separated by electrophoresis on 12% (w/v) polyacrylamide gels (SDS-PAGE), and transferred to polyvinylidene fluoride membranes (Millipore Corporation, Bedford, MA) in a semi-dry transfer device (Biometra, Göttingen, Germany). Membranes were incubated for 60 min in TBS, pH 7.4, containing 0.1% (v/v) Tween-20 (TBS-T) and 5% (w/v) dried skimmed milk, followed by an overnight incubation with the polyclonal rab31-directed chicken or rabbit antibodies, diluted in TBS-T supplemented with 5% (w/v) dried skimmed milk. After washing with TBS-T, binding of the antibodies was visualized by incubation of the membranes with a secondary HRP-conjugated goat anti-rabbit IgG (Jackson ImmunoResearch Lab) or an HRP-labeled rabbit anti-chicken IgY (Sigma-Aldrich), followed by chemiluminescent reaction using ECL (Thermo Scientific, Pierce).

### ELISA for quantitative assessment of rab31

For detection of rab31 in cell lysates, a sandwich ELISA format was developed using a commercial monoclonal antibody (mAb) to rab31 (mAb M01, Novus Biologicals, Inc., Littleton, CO) as catcher antibody and own polyclonal antibody (pAb) from rabbit #3 (RT3-IgG) as detecting antibody. Recombinant, purified rab31-His protein, dialyzed against PBS, pH 7.4, containing 1 mM DTT, was used as the reference antigen (stock 20 μg/ml). Ninety-six-well polystyrene plates (MaxiSorp^TM^; Thermo Scientific, Nunc) were coated overnight at 4°C with mAb M01 (clone 1C6; Novus Biologicals) diluted in coating buffer (15 mM Na_2_CO_3_, 33 mM NaHCO_3_, pH 9.6). After washing of the plates twice with washing buffer (PBS, containing 0.5% [v/v] Tween 20, pH 7.6), wells were blocked with blocking solution (washing buffer containing 2% [v/v] neonatal calf serum; Gibco BRL) for 30 min at 37°C. Thereafter, plates were incubated with cell lysates diluted in sample buffer (50 mM Tris-HCl, 100 mM NaCl, containing 0.2% [v/v] Triton X-100, and 0.2% [w/v] BSA, pH 7.6) for 90 min at 37°C. Two-fold serial dilutions of recombinant rab31-His protein in sample buffer, covering a concentration range of 0.15 to 5 ng/ml, were employed to construct a standard curve. After washing, wells were incubated with pAb RT3-IgG for 90 min at 37°C followed by incubation with secondary HRP-labeled goat anti-rabbit IgG (Novus Biologicals) for 60 min at 37°C. Finally, plates were washed and the peroxidase reaction initiated by addition of TMB / H_2_O_2_ (K & P Laboratories, Gaithersburg, MD) as the peroxidase substrate. After 20 min at RT, the reaction was stopped by addition of 0.5 M H_2_SO_4_. The optical density of the resulting color was determined at 450 nm (reference wavelength 620 nm) using an automated multichannel ELISA reader. Rab31 antigen levels are given as ng per mg of total protein.

### Immunohistochemistry

Full-face sections from invasive ductal breast cancer tissue specimens were selected for immunostaining with pAb RT3-IgG and pAb from animal #4 (RT4-IgG), respectively. Formalin-fixed, paraffin-embedded breast cancer tissue specimens were obtained from archival material of the Institute of Pathology, Dresden University of Technology. The study adhered to national regulations on ethical issues and was approved by the local ethics committee at the Dresden University Medical Center. Immunohistochemical staining was performed as described previously with minor modifications [19,53]. Briefly, tissue sections were dewaxed, rehydrated, and treated for antigen retrieval by pressure cooking (15 min at 120°C) in 100 mM citrate buffer, pH 6.0. After several washes with PBS, sections were treated with 0.3% H_2_O_2_ for 10 min at RT to block endogenous peroxidase activity. Normal serum diluted in PBS was applied for 45 min at RT to block nonspecific antibody binding. Subsequently, primary antibodies (dilution: 1:2,000) were allowed to react overnight at 4°C followed by incubation with biotinylated anti-rabbit IgG (Vectastain Elite ABC Kit, Vector Laboratories, Burlingame, CA) for 50 min at RT. After washing, the Vectastain Elite ABC-reagent was applied for 50 min at RT, and the washing steps were repeated. The peroxidase reaction was developed with 3,3’-diaminobenzidine (Sigma-Aldrich) for 10 min at RT. Finally, counterstaining of nuclei was performed with hematoxylin. As a negative control, the primary antibody was omitted and replaced by PBS or by an irrelevant antibody.

### Cell-based in vitro assays

#### Proliferation assays

Transfected MDA-MB-231 (and MDA-MB-435) cells were seeded in 24-well microtiter plates at a density of 20,000 cells per well, CAMA-1 cells at a density of 30,000 cells per well. All cell lines were incubated for 24, 48, 72 or 96 h at 37°C. After incubation, cells were detached by PBS plus 0.05% (w/v) EDTA (Biochrom AG, Berlin, Germany). Living cells were identified by trypan blue exclusion and counted in a hemocytometer under a light microscope.

#### Cell adhesion assays

Ninety-six-well plates were coated for 1 h at RT with vitronectin (2 μg/ml) or fibronectin (5 μg/ml) (BD Biosciences, Franklin Lakes, NJ), or for 3 h at 37°C with 5 μg/well of laminin, collagen type I or collagen type IV (Sigma-Aldrich), all diluted in PBS. Cells were resuspended in culture medium containing 0.5% (w/v) BSA and HEPES (Invitrogen), were seeded at a density of 20,000 cells/well (MDA-MB-231 and MDA-MB-435) or 30,000 cells/well (CAMA-1), and allowed to adhere to the extracellular matrix proteins for 2 h at 37°C. Subsequently, non-adherent cells were removed by washing three times with PBS. The number of adherent cells was quantified by a hexosaminidase activity assay. For this, cells were incubated with p-nitrophenyl-N-acetyl-β-D-glucosaminide (Sigma-Aldrich) diluted to 15 mM in 100 mM sodium citrate, pH 5.0, containing 0.5% (v/v) Triton X-100 for 90 min at 37°C. The reaction was terminated by the addition of stop buffer (0.2 M NaOH, 5 mM EDTA) and the optical density recorded at 405 nm using an automated ELISA reader.

#### Cell invasion assays

Invasion assays were performed using transwell inserts (8 μm pore size; Corning Costar, Amsterdam, The Netherlands). Thirty μg of basement membrane complex growth factor reduced Matrigel^TM^ (BD Biosciences) was diluted in 100 μl cold FCS-free DMEM, containing 0.1% (w/v) BSA and applied to the upper side of the insert. After 3 h incubation at 37°C, followed by overnight incubation in a laminar hood at RT, inserts were rehydrated with 200 μl FCS-free DMEM, containing 0.1% (w/v) BSA for 2 h at 37°C. Cells suspended in culture medium were seeded into the upper chamber of the device at a density of 40,000 cells/chamber. The lower chambers were filled with 600 μl DMEM supplemented with 10% (v/v) FCS as the chemoattractant. After 24 h incubation at 37°C, Matrigel^TM^ and non-invaded cells, located on the upper side of the insert, were removed with a Q-tip, whereas invaded cells on the lower side of the insert were fixed, stained using Hemacolor (Merck, Darmstadt, Germany), and counted under a light microscope.

### Experimental metastasis assay

MDA-MB-231 cells are tumorigenic and invasive cells, which upon intravenous injection in mice colonize to the lungs [54]. Transfected MDA-MB-231 cells were genetically tagged with the *lacZ-*gene, allowing X-Gal staining, as reported previously [55]. Pathogen-free, 8 to 10 weeks old female athymic (nu/nu) mice were obtained from Charles River Laboratories (Sulzfeld, Germany). Mice were allocated to two groups and 1 × 10^6^ control MDA-MB-231-vector cells (n = 7) or pRcRSV-rab31-transfected MDA-MB-231 (batch) cells (n = 7) intravenously inoculated into the tail vein. At day 35 post-injection animals were sacrificed, lungs isolated and the *lacZ*-tagged tumor cells stained with 5-bromo-4-chloro-indolyl-β-D-galactopyranoside (X-Gal) (Roche Diagnostics, Mannheim, Germany) as described [56]. Thereafter, blue stained cellular foci were counted as metastases. All animal experiments were done in compliance with the guidelines and with confirmed ethical approval of the *Tierschutzgesetz des Landes Bayern (Regierung von Oberbayern).*

### Statistical analyses

Group differences and p-values were calculated using the Mann-Whitney U-test. P values < 0.05 were considered as statistically significant.

## Abbreviations

GST-rab31: Glutathione S-transferase-rab31 fusion protein; rab31-ΔCC: rab31 with a deletion of two C-terminal cysteines; rab31-His: rab31 histidine-tag fusion protein; rab31-Q64L: rab31 with an exchange of glutamine 64 by leucine; RT3-IgG: rabbit animal #3-IgG; RT4-IgG: rabbit animal #4-IgG; uPAR-del4/5: uPAR splice variant.

## Competing interests

The authors declare that they have no competing interests.

## Authors’ contributions

VM conceived the study, made substantial contributions to the conception and design of the study, and drafted the manuscript. BG participated in the design of the study, generated rab31 overexpressing cell lines, isolated cell clones, carried out cell-based *in vitro* assays, participated in the characterization of antibodies and in the *in vivo* experiment, and has been substantially involved in drafting the manuscript. SSö generated rab31(-mutant) overexpressing cell lines and carried out cell-based *in vitro* assays. SSch produced and purified the rab31 immunogen and was involved in the characterization of antibodies by Western blot and microtiter plate-based assays. BS performed the experimental *in vivo* metastasis assay. TK participated in the characterization of antibodies and carried out the immunoassay experiments. TL and MK were involved in the design and coordination of the study, participated in the characterization of antibodies and its application in immunoassays and immunohistochemistry, and have been substantially involved in drafting the manuscript. AK supervised the *in vivo* experiment and has been involved in drafting and critically revising the manuscript. GB and MS participated in the design of the study and have been involved in drafting and critically revising the manuscript. All authors have read and approved the final version of the manuscript.

## Supplementary Material

Additional file 1 **Figure S1. **Analysis of MDA-MB-231 cell transfectants for rab31 expression by immunofluorescence. After overnight growth on fibronectin-coated glass slides, the cell monolayers were fixed and permeabilized with PBS containing 4% (w/v) paraformaldehyde, 0.025% (w/v) saponin. After incubation, the rabbit antibody directed to rab31 was detected with Alexa488-labeled goat anti-rabbit IgG (Sigma-Aldrich) and fluorescence signal intensity visualized by confocal laser scanning microscopy (CLSM). Typical fluorescent images (lower panel) together with the corresponding differential interference contrast images (upper panel) are depicted. In immunocytochemistry, a often pronounced perinuclear staining is visible. There are no apparent morphological differences between vector control cells and cells overexpressing rab31. Click here for file

Additional file 2 ** Figure S2.** The constitutively active rab31-mutant Q64L, but not the inactive form rab31-ΔCC, induces cell growth and reduces cell adhesion, when overexpressed in MDA-MB-231 breast cancer cells. **(A) Overexpression of rab31 mutants in breast cancer cells.** MDA-MB-231 cells were stably transfected with rab31 expression plasmids encoding either the constitutively active mutant Q64L, the inactive form rab31-ΔCC, or the empty vector pRcRSV only. Western blot analysis demonstrated low endogenous rab31 expression in vector control cells, whereas the cells overexpressing either of the rab31 mutants display strongly elevated, but similar rab31-reactive protein levels. **(B) Overexpression of rab31-Q64L, but not rab31-ΔCC, enhances cell growth of breast cancer cells.** Stably transfected MDA-MB-231 cells were seeded in triplicate onto 24-well plates, detached with 0.1% EDTA-solution after 24 and 96 h of cultivation and counted with a Neubauer hemocytometer under trypan blue exclusion. Cell number at 24 h was set to 100%; increase in cell number was expressed relative to the 24 h value in %. Mean values (± SEM) of seven independent experiments are depicted. Statistically significant differences (p ≤ 0.01) are indicated by an asterisk. **(C) Overexpression of rab31-Q64L, but not rab31-ΔCC, reduces the adhesive capacity of breast cancer cells.** Stably transfected MDA-MB-231 cells were seeded on collagen type IV-coated 96-well plates. After 2 h of cell cultivation, the number of adherent cells was monitored by the hexosaminidase activity assay as described in the Methods section. Seven independent experiments were performed in triplicates each. The results are given in % relative to the cell number of adherent vector-transfected control cells. Whisker box plots indicate the 25th and 75th percentile, the vertical bars indicate the 10th and 90th percentile. The median value of seven experiments is indicated by a bar within the box. Statistically significant differences (p < 0.01) are indicated by an asterisk. Similar effects were observed with the extracellular matrix proteins collagen type I, laminin, vitronectin, and fibronectin (data not shown). Click here for file

## Author details

^1^Clinical Research Unit, Department of Obstetrics and Gynecology, Technical University of Munich, Ismaninger Str. 22, Munich 81675, Germany. ^2^Institute of Experimental Oncology and Therapy Research, Technical University of Munich, Ismaninger Str. 22, Munich 81675, Germany. ^3^Medizinisches Labor Ostsachsen, Töpferstr. 17, Bautzen, 02625, Germany. ^4^Institute of Pathology, Dresden University of Technology, Fetscherstr. 74, Dresden 01307, Germany.
