# Supplementary figures and images for: Rab31 expression levels modulate tumor-relevant characteristics of breast cancer cells
Source: Mol Cancer. 2012 Aug 24;11:62. doi: 10.1186/1476-4598-11-62 (PMC3499445; doi:10.1186/1476-4598-11-62)

## MDA-MB-231 cells

---

vector control

rab31 high

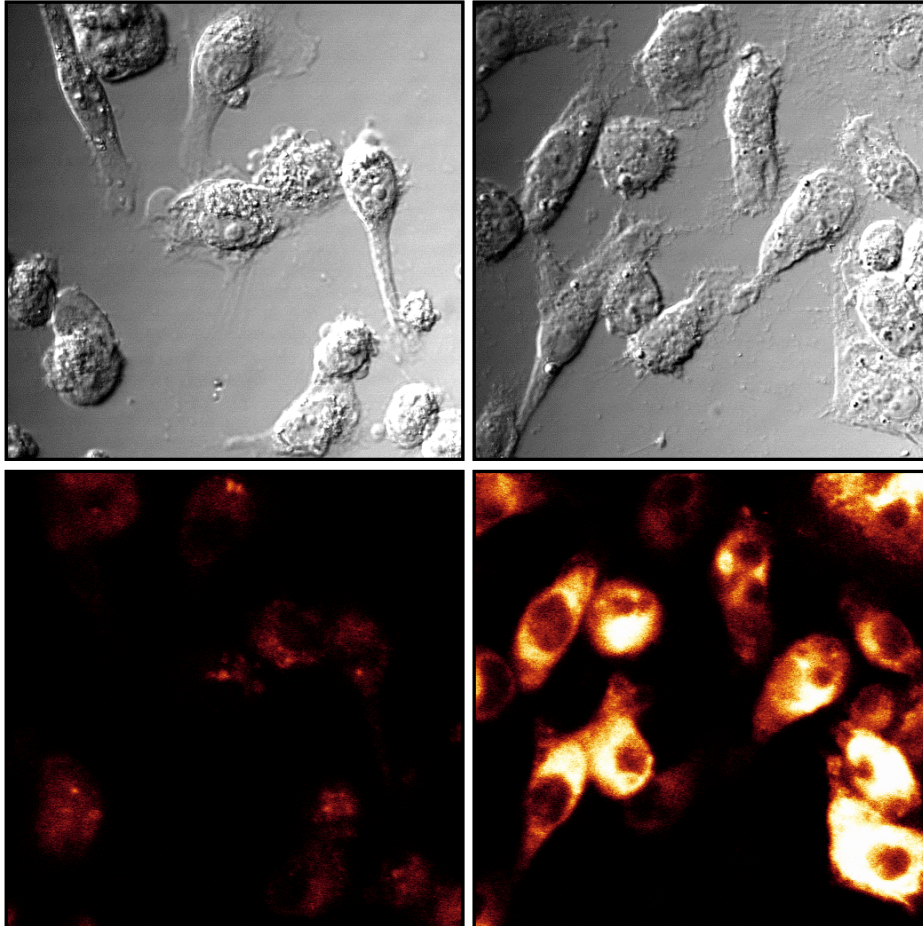

Suppl. Figure 1

Supplement: Additional file 1 — Figure S1. Analysis of MDA-MB-231 cell transfectants for rab31 expression by immunofluorescence. After overnight growth on fibronectin-coated glass slides, the cell monolayers were fixed and permeabilized with PBS containing 4% (w/v) paraformaldehyde, 0.025% (w/v) saponin. After incubation, the rabbit antibody directed to rab31 was detected with Alexa488-labeled goat anti-rabbit IgG (Sigma-Aldrich) and fluorescence signal intensity visualized by confocal laser scanning microscopy (CLSM). Typical fluorescent images (lower panel) together with the corresponding differential interference contrast images (upper panel) are depicted. In immunocytochemistry, a often pronounced perinuclear staining is visible. There are no apparent morphological differences between vector control cells and cells overexpressing rab31. [file 1476-4598-11-62-S1.pdf]

**A**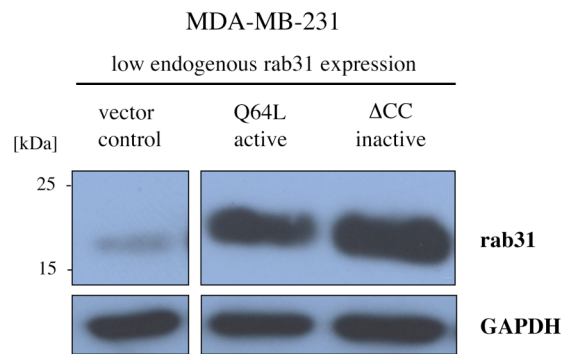**B**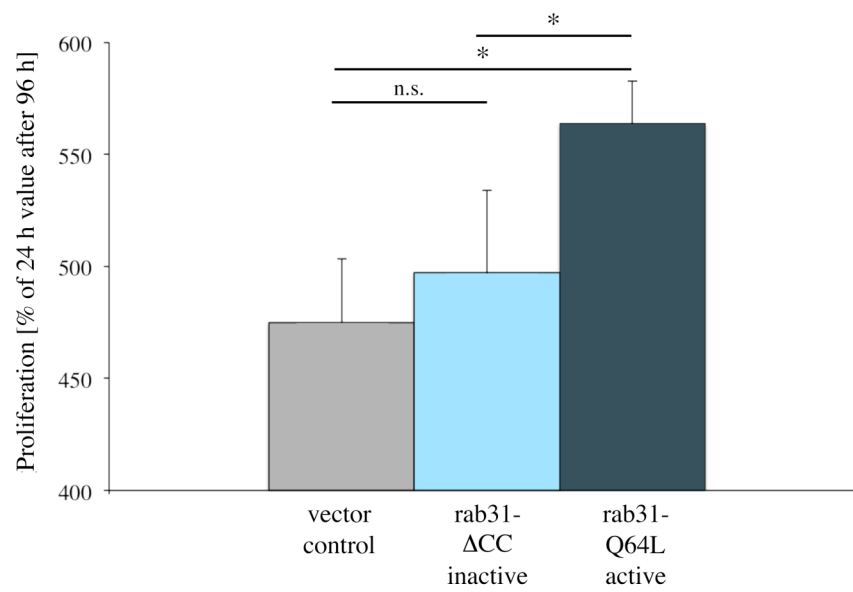**C**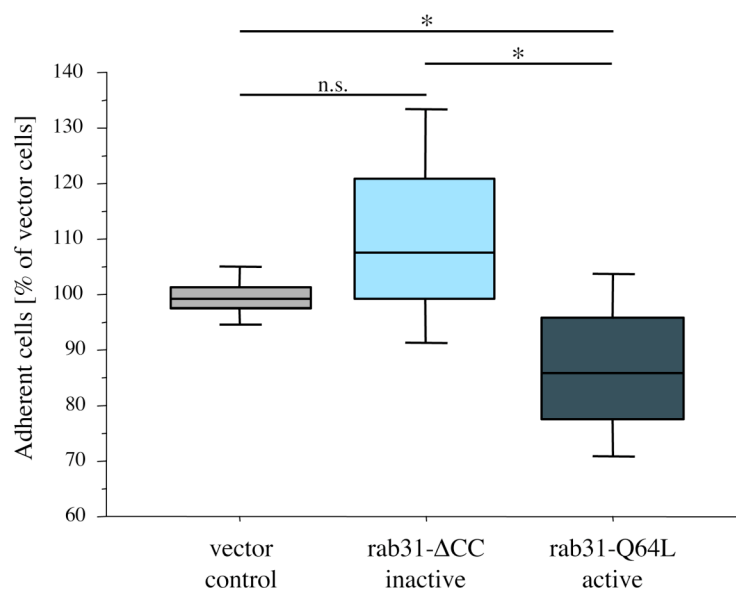

Supplement: Additional file 2 — Figure S2. The constitutively active rab31-mutant Q64L, but not the inactive form rab31-ΔCC, induces cell growth and reduces cell adhesion, when overexpressed in MDA-MB-231 breast cancer cells. (A) Overexpression of rab31 mutants in breast cancer cells. MDA-MB-231 cells were stably transfected with rab31 expression plasmids encoding either the constitutively active mutant Q64L, the inactive form rab31-ΔCC, or the empty vector pRcRSV only. Western blot analysis demonstrated low endogenous rab31 expression in vector control cells, whereas the cells overexpressing either of the rab31 mutants display strongly elevated, but similar rab31-reactive protein levels. (B) Overexpression of rab31-Q64L, but not rab31-ΔCC, enhances cell growth of breast cancer cells. Stably transfected MDA-MB-231 cells were seeded in triplicate onto 24-well plates, detached with 0.1% EDTA-solution after 24 and 96 h of cultivation and counted with a Neubauer hemocytometer under trypan blue exclusion. Cell number at 24 h was set to 100%; increase in cell number was expressed relative to the 24 h value in %. Mean values (± SEM) of seven independent experiments are depicted. Statistically significant differences (p ≤ 0.01) are indicated by an asterisk. (C) Overexpression of rab31-Q64L, but not rab31-ΔCC, reduces the adhesive capacity of breast cancer cells. Stably transfected MDA-MB-231 cells were seeded on collagen type IV-coated 96-well plates. After 2 h of cell cultivation, the number of adherent cells was monitored by the hexosaminidase activity assay as described in the Methods section. Seven independent experiments were performed in triplicates each. The results are given in % relative to the cell number of adherent vector-transfected control cells. Whisker box plots indicate the 25th and 75th percentile, the vertical bars indicate the 10th and 90th percentile. The median value of seven experiments is indicated by a bar within the box. Statistically significant differences (p < 0.01) are indica [file 1476-4598-11-62-S2.pdf]
